# Pharmacokinetics and Biodistribution of a Human Monoclonal Antibody to Oxidized LDL in Cynomolgus Monkey Using PET Imaging

**DOI:** 10.1371/journal.pone.0045116

**Published:** 2012-09-17

**Authors:** Amrita V. Kamath, Simon P. Williams, Sherry Bullens, Kyra J. Cowan, Yvonne Stenberg, Simon R. Cherry, Stephen Rendig, David L. Kukis, Chris Griesemer, Lisa A. Damico-Beyer, Stuart Bunting

**Affiliations:** 1 Genentech Research and Early Development, Genentech, Inc., South San Francisco, California, United States of America; 2 Bioanalytical and Protein Chemistry, BioInvent International AB, Lund, Sweden; 3 Center for Molecular and Genomic Imaging, University of California Davis, Davis, California, United States of America; University of Milan, Italy

## Abstract

**Purpose:**

Oxidized low-density lipoprotein (LDL) plays an essential role in the pathogenesis of atherosclerosis. The purpose of this study was to characterize the pharmacokinetics (PK) of a human recombinant IgG1 antibody to oxidized LDL (anti-oxLDL) in cynomolgus monkey. The tissue biodistribution of anti-oxLDL was also investigated using positron emission tomography (PET) imaging.

**Methods:**

Anti-oxLDL was conjugated with the N-hydroxysuccinimide ester of DOTA (1,4,7,10-tetraazacyclododecane 1,4,7,10-tetraacetic acid) and radiolabeled by chelation of radioactive copper-64 (^64^Cu) for detection by PET. Anti-oxLDL was administered as a single intravenous (IV) dose of 10 mg/kg (as a mixture of radiolabeled and non-labeled material) to two male and two female cynomolgus monkeys. Serum samples were collected over 29 days. Two ELISA methods were used to measure serum concentrations of anti-oxLDL; Assay A was a ligand binding assay that measured free anti-oxLDL (unbound and partially bound forms) and Assay B measured total anti-oxLDL. The biodistribution was observed over a 48-hour period following dose administration using PET imaging.

**Results:**

Anti-oxLDL serum concentration-time profiles showed a biphasic elimination pattern that could be best described by a two-compartment elimination model. The serum concentrations obtained using the two ELISA methods were comparable. Clearance values ranged from 8 to 17 ml/day/kg, while beta half-life ranged from 8 to12 days. The initial volume of distribution and volume of distribution at steady state were approximately 55 mL/kg and 150 mL/kg, respectively. PET imaging showed distribution predominantly to the blood pool, visible as the heart and great vessels in the trunk and limbs, plus diffuse signals in the liver, kidney, spleen, and bone marrow.

**Conclusions:**

The clearance of anti-oxLDL is slightly higher than typical IgG1 antibodies in cynomolgus monkeys. The biodistribution pattern appears to be consistent with an antibody that has no large, rapid antigen sink outside the blood space.

## Introduction

Atherosclerosis is the development of plaque in the inner layer of the artery and is a major cause of acute myocardial infarction, stroke and peripheral artery disease [1], [2]. Human apolipoprotein B-100 (ApoB-100) is the protein component of low-density lipoprotein (LDL) which is the main carrier of cholesterol in circulation. Oxidized LDL (oxLDL) plays an essential role in the pathogenesis of atherosclerosis, vascular inflammation and related metabolic disorders [1], [3]. Oxidation of LDL leads to its conversion to an atherogenic particle, and the oxidative modifications drive the initial formation of fatty streaks, the earliest visible atherosclerotic lesions. There is a strong link between levels of oxidized lipoproteins and inflammatory processes that lead to the formation of atherosclerotic plaques in arterial walls [1], [3]. OxLDL is thought to promote atherosclerosis through complex inflammatory and immunologic mechanisms that lead to lipid dysregulation, foam cell formation, and monocyte/macrophage activation [3]. OxLDL binds to scavenger receptors on macrophages present in plaques, leading to activation and release of proteins such as monocyte chemoattractant protein 1 (MCP-1) from the macrophages, which recruit new monocytes into the plaque that subsequently become activated, leading to an aggravated state of inflammation [3]. Atherosclerotic plaque inflammation is critical to the pathophysiology of acute coronary syndrome (ACS), and oxLDL is thought to be a key mediator of this process [4]–[6].

Anti-oxLDL is a fully human monoclonal immunoglobulin G1 (IgG1) antibody targeted to oxidized human ApoB-100. Anti-oxLDL is designed to interfere with the inflammation cascade within the plaque, decreasing the activity of pro-inflammatory cells and causing plaques to stabilize. More stable plaques are less likely to rupture, an event that leads to blood clot formation and which can completely block blood flow to the heart, causing a heart attack. Anti-oxLDL has been shown to reduce plaque formation in in vivo mouse models of atherosclerosis [3], [7]. Several in vivo and in vitro studies have shown inhibition of macrophage recruitment and pro-inflammatory activity as important mechanisms underlying the activity of anti-oxLDL [3], [7]. Anti-oxLDL is being developed as a potential therapeutic for the secondary prevention of major cardiac events in high risk patients with ACS. In vitro studies using BiaCore have shown that anti-oxLDL binds with high affinity to the oxidized form of LDL (oxLDL) in both humans and cynomolgus monkeys (data not shown). Since it binds to oxLDL in cynomolgus monkeys and humans with similar affinity, the cynomolgus monkey was determined to be an appropriate species to characterize the pharmacokinetics of anti-oxLDL. In addition, cynomolgus monkey is an animal model that is used extensively in the characterization of monoclonal antibody pharmacokinetics prior to clinical use, because this species is closely related, both phylogenetically and physiologically, to humans. In this report we have characterized the pharmacokinetics of anti-oxLDL in cynomolgus monkey and investigated its gross tissue biodistribution using positron emission tomography (PET) imaging.

## Materials and Methods

### Materials

A mixture of radiolabeled anti-oxLDL and non-radiolabeled anti-oxLDL was used in this study. Non-radiolabled anti-oxLDL was generated at Genentech, Inc., and was supplied in a clear yellow liquid at a concentration of 98.9 mg/mL (pH 5.5) for the study. To prepare radiolabeled anti-oxLDL, the antibody was conjugated with the N-hydroxysuccinimide ester of DOTA (1,4,7,10-tetraazacyclododecane 1,4,7,10-tetraacetic acid) and radiolabeled by chelation of radioactive copper-64 (^64^Cu) to be detectable by positron emission tomography (PET) as described previously [8], [9]. The DOTA molecule is a metal chelator that enables radiometal chelation to the antibody moiety. Two batches of DOTA conjugated material were prepared (∼2.5 DOTA per antibody) and pooled together to get final conjugated material with a concentration of 11.4 mg/mL. Binding activity of both batches of conjugated material was tested by an ELISA method to see that DOTA conjugation did not significantly affect the binding of anti-oxLDL to its antigen. On the day of dosing, DOTA-anti-oxLDL was radiolabeled with ^64^Cu. Purified ^64^Cu-DOTA-anti-oxLDL was then formulated with unmodified anti-oxLDL and phosphate buffered saline (PBS), and sterilized by filtration, to prepare a dose solution of 6 mCi/120 mg/6 mL. Thin-layer chromatography indicated 100% radiochemical purity.

### Evaluation of Anti-oxLDL Binding Activity after DOTA Conjugation

To show that DOTA conjugation did not affect the binding of anti-oxLDL to its antigen, the binding activity of both batches of DOTA conjugated anti-oxLDL were compared with that of two different lots of unconjugated anti-oxLDL using an ELISA. MDA-ApoB100 (100 µg/mL) was used as the capture reagent and 130 ng/mL rabbit anti-HuIgG conjugated to horseradish peroxidase (HRP; Dako North America, Inc.; Carpinteria, CA) was used as the detection reagent. Results were compared with a standard curve that covered a range of 1−2190 ng/mL anti-oxLDL.

### Study Design and In-Life Study in Cynomolgus Monkeys

#### Ethics statement

In vivo imaging and pharmacokinetic studies in cynomolgus monkeys were conducted at the California National Primate Research Center (CNPRC) at the University of California, Davis (UC Davis) according to all Federal, State and local guidelines for the use of animals in research, and in compliance with Animal Welfare Assurance A3433-01 on file at the NIH. Protocols were reviewed and approved by the Institutional Animal Care and Use Committees at both Genentech (Protocol number GNE03/07-1271) and UC Davis (Protocol number 07-12965). The studies involved imaging and collection of blood samples, neither of which required procedures that would cause more than slight or momentary pain or distress to the animals. All injections, intubations and catheter placements were conducted under anesthesia by qualified primate therapeutic staff at the CNPRC. Imaging was conducted under general anesthesia and the animals were monitored continuously by the same qualified staff during imaging, blood draws and during recovery from anesthesia. Following recovery from anesthesia, all animals were returned to the colony, where they are subject to regular follow-up health checks by veterinary staff at the CNPRC. Provisions were made in the approved protocol for veterinary intervention in the case of any distress or morbidity from the injected agent, anesthesia, imaging, or blood draws, however no adverse events occurred during the studies or during the recovery period.

#### Study design in cynomolgus monkeys

Two male and 2 female cynomolgus monkeys were given a single IV bolus dose of a mixture of ^64^CU-DOTA-anti-oxLDL and anti-oxLDL, which yielded a total anti-oxLDL dose of approximately 10 mg/kg (approximately 1.5 mCi/animal) via a catheter inserted into a peripheral vein. For biodistribution analyses, PET imaging was performed on 2 out of 4 animals at 1 hour post-dose and on all 4 animals at 24 and 48 hours post-dose. Blood samples for PK and anti-therapeutic antibody (ATA) analyses were drawn from all animals before dose administration and at 5 minutes, 1 and 24 hours, and 4, 8, 15, and 29 days post-dose and processed for serum. The age of the four normal healthy monkeys on study ranged from 3 years 2 months to 7 years 10 months (considered to be young animals).

### Evaluation of Anti-oxLDL Concentrations in Serum

Serum samples from cynomolgus monkeys were analyzed for anti-oxLDL concentrations using two quantitative enzyme-linked immunosorbent assays (ELISA); Assay A was a ligand biding assay that measured free anti-oxLDL, which included unbound and partially bound forms of anti-oxLDL, while Assay B measured total anti-oxLDL, which included unbound, partially bound, and bound forms of anti-oxLDL. Assay A was a specific-coat assay that used malondialdehyde (MDA) oxidized ApoB100 (Academy BioMedical Co.; Houston, TX) as the capture reagent and a rabbit anti−human IgG− HRP (Dako) as the detection reagent. Assay B used a sheep anti−human IgG coat as the capture reagent and a sheep anti−human IgG−HRP as a detection reagent. The minimum quantifiable concentration in both assays was 400 ng/mL.

### Anti-therapeutic Antibody (ATA) Analysis

An assay developed on the Meso Scale Discovery (MSD; Gaithersburg, Maryland) electrochemiluminescence assay (ECLA) platform was used to detect antibodies against anti-oxLDL in serum. Serum samples were diluted 1∶50 in assay diluent (PBS with 0.5% polysorbate 20 and 0.05% proclin 300) and incubated with optimized levels of drug conjugates (biotin−anti-oxLDL and ruthenium−anti-oxLDL) overnight at room temperature. Sample mixtures were added to blocked streptavidin-coated MSD plates, incubated, and then analyzed for electrochemiluminescent units (ECLU). The initial cutpoint factor was determined based on the ECLU of samples from 25 cynomolgus monkeys, relative to a negative pool of normal cynomolgus monkey serum. A sheep anti−human affinity purified IgG, diluted in pooled cynomolgus monkey serum, was used as the positive control. The assay sensitivity was determined to be 422 ng/mL and test-article (anti-oxLDL) tolerance was 109 µg/mL.

### Pharmacokinetic Data Analysis

Serum concentration-time profiles were used to estimate the PK parameters in cynomolgus monkeys using WinNonlin (version 5.1.1; Pharsight Corporation, Mountain View, CA). A two-compartment elimination model with IV bolus input and first-order elimination was used to describe the observed data and the following PK parameters were reported: total drug exposure defined as area under the curve from the serum concentration−time profiles extrapolated to infinity (AUCinf), clearance (CL), predicted maximum serum concentration (Cmax), volume of distribution of the central compartment (V1), volume of distribution at steady state (Vss), half-life associated with the distribution phase (T1/2α), and half-life associated with the elimination phase (T1/2β). Data generated from each of the assays (Assay A and B) for each animal were analyzed separately, and results for each assay were summarized as mean ± standard deviation (SD).

### PET Imaging and Data Analysis

PET scanning was performed using a dedicated primate imaging system, the microPET™ P4 (Concorde Microsystems; Knoxville, TN; now part of Siemens Medical Solutions; Hoffman Estates, IL). At selected time points, PET imaging data from each of three contiguous bed positions were combined to give neck-to-pelvis anatomical coverage in each animal. Images were reconstructed for each bed position using an iterative maximum a posteriori algorithm and fused into a single three dimensional (3D) data set, using software provided with the microPET™ system. There was no overlap in the images for adjacent bed positions. The 3D images were displayed and quantified using Analyze™ software (AnalyzeDirect; Lenexa, KS).

## Results

### Evaluation of Anti-oxLDL Binding Activity after DOTA Conjugation

**Figure 1:**
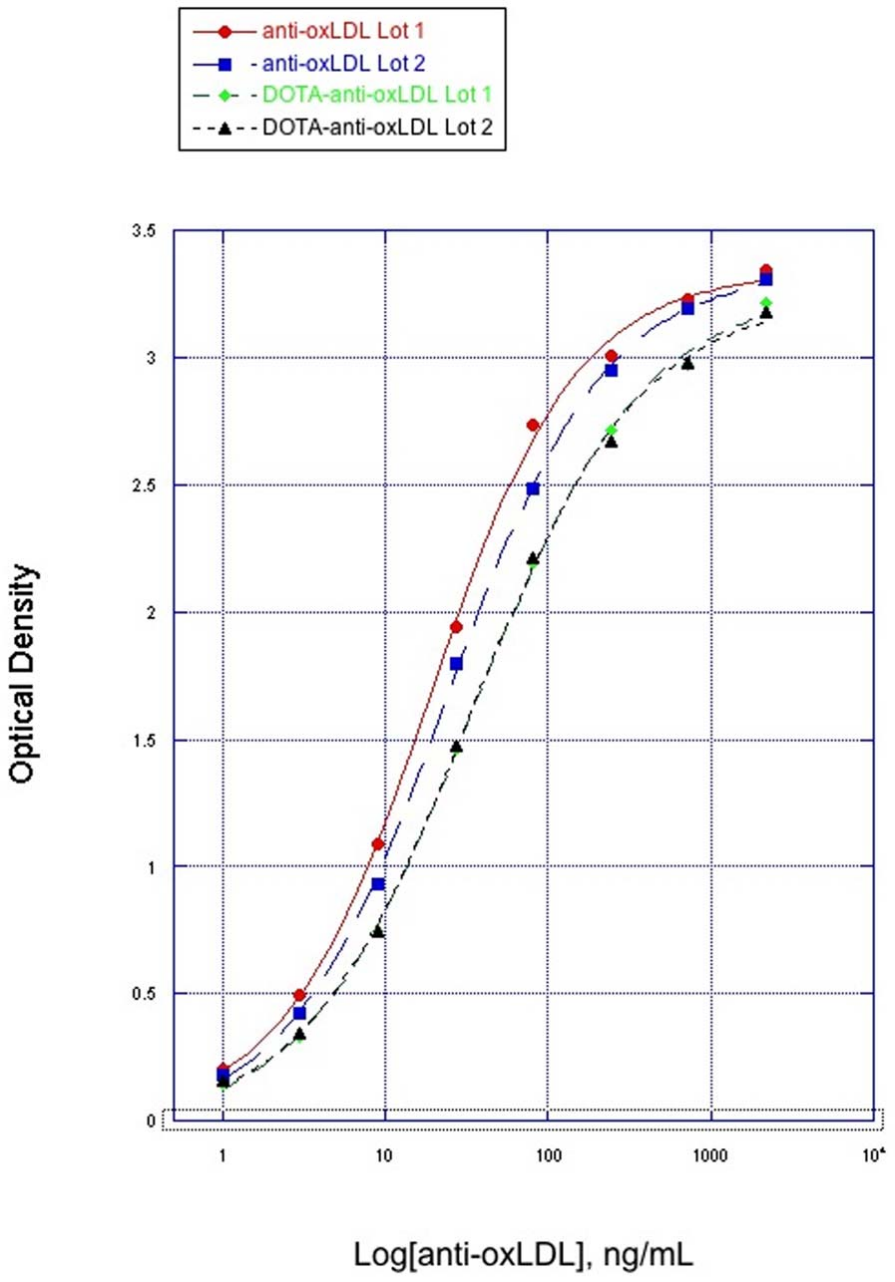
Binding of anti-oxLDL (conjugated and unconjugated) to MDA-ApoB100.

The ELISA compared two lots of unconjugated anti-oxLDL with two batches of DOTA conjugated anti-oxLDL. The EC50 values of unconjugated anti-oxLDL ranged from 19.4–24.8 ng/mL. After conjugation with DOTA, the EC50 values of DOTA-anti-oxLDL ranged from 34.7–36.1 ng/mL. These data indicate that the DOTA conjugated anti-oxLDL had slightly reduced binding to Apo-100 compared to the unconjugated anti-oxLDL. The binding curves of both the unconjugated antibody and the DOTA conjugated antibody are shown in Figure 1.

### Anti-Therapeutic Antibody (ATA) Analysis

Only 1 animal (animal 2 M) out of the 4 animals in the study had a detectable ATA signal. Samples from this animal produced a low-level positive signal at the predose timepoint which persisted throughout the dosing period. As this signal was detected at the predose timepoint, it was not considered test-article induced and could represent some other factor that could bridge the ATA assay. Samples from the remaining three animals did not have detectable levels of ATAs. However, since the anti-oxLDL tolerance was high in the ATA assay (109 µg/mL), it is possible that there could have been some interference by anti-oxLDL with the detection by of ATAs.

### Pharmacokinetics in Cynomolgus Monkeys

**Figure 2:**
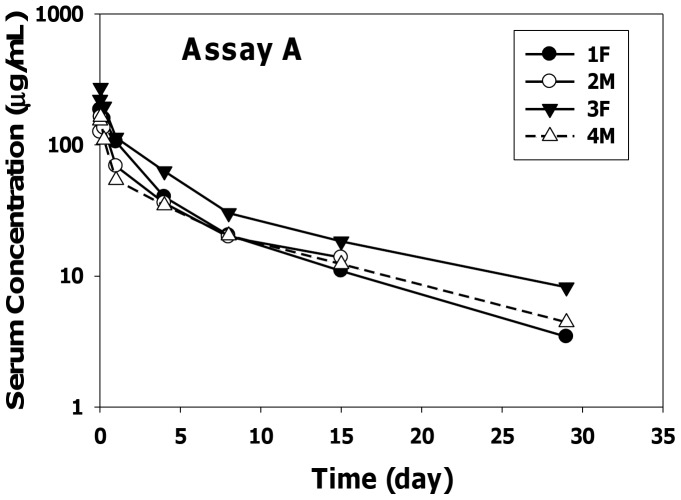
Anti-oxLDL Individual Serum Concentration−Time Profiles Measured by Assay A.

**Figure 3:**
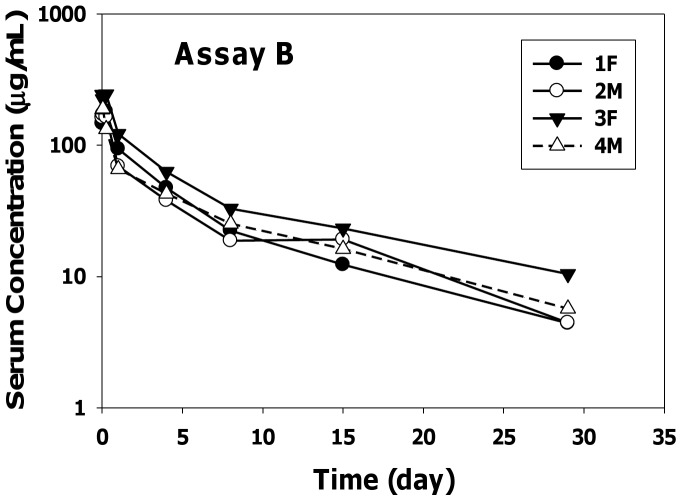
Anti-oxLDL Individual Serum Concentration-Time Profiles Measured by Assay B.

The serum-concentration profiles of anti-oxLDL in cynomolgus monkeys using two different ELISA methods following a single IV bolus dose of 10 mg/kg are shown in Figure 2 (Assay A) and Figure 3 (Assay B). The PK parameters after two-compartmental analysis using assays A and B are summarized in Tables 1 and 2, respectively. Following dose administration, anti-oxLDL showed a biphasic elimination pattern that could be best described by a two-compartment elimination model.

**Table 1:** PK Parameters following IV Administration of a Single Dose of 10 mg/kg of Anti-oxLDL (Assay A).

Animal ID (Sex)	AUCinf(day•µg/mL)	CL(mL/day/kg)	T1/2α(day)	T1/2β(day)	Cmax(µg/mL)	V1(mL/kg)	Vss(mL/kg)
1 (F)	681	14.7	1.13	8.37	177	56.6	126
2 (M)	642	15.3	0.565	8.75	153	64.0	168
3 (F)	1102	9.44	1.30	11.8	229	45.5	116
4 (M)	591	17.4	0.324	8.68	163	63.1	198
Mean	754	14.2	0.831	9.40	180	57.3	152
SD	235	3.39	0.462	1.60	33.5	8.52	38.2

SD: standard deviation.

**Table 2:** PK Parameters following IV Administration of a Single Dose of 10 mg/kg of Anti-oxLDL (Assay B).

Animal ID (Sex)	AUCinf(day•µg/mL)	CL(mL/day/kg)	T1/2α(day)	T1/2β(day)	Cmax(µg/mL)	V1(mL/kg)	Vss(mL/kg)
1 (F)	758	13.2	1.42	9.58	163	61.4	129
2 (M)	718	13.7	0.511	8.88	180	54.5	153
3 (F)	1250	8.34	1.13	12.5	239	43.4	117
4 (M)	748	13.8	0.323	8.91	195	52.9	162
Mean	868	12.2	0.846	9.98	194	53.0	140
SD	253	2.61	0.515	1.74	32.7	7.37	20.6

SD: standard deviation.

Overall the serum concentrations and pharmacokinetic parameters obtained using the two ELISA methods were fairly similar. The clearance values ranged from 8 to 17 mL/day/kg and beta half-life ranged from 8 to 12 days. The initial volume of distribution approximated the serum volume of monkeys and the volume of distribution at steady state was approximately three-fold the V1 values. The positive signal in the ATA assay that was detected in 1 of 4 animals given anti-oxLDL had minimal impact on the PK parameter estimates. In addition, the PK parameters in female and male cynomolgus monkeys were similar suggesting no gender differences in the pharmacokinetics of anti-oxLDL.

### PET Imaging

**Figure 4:**
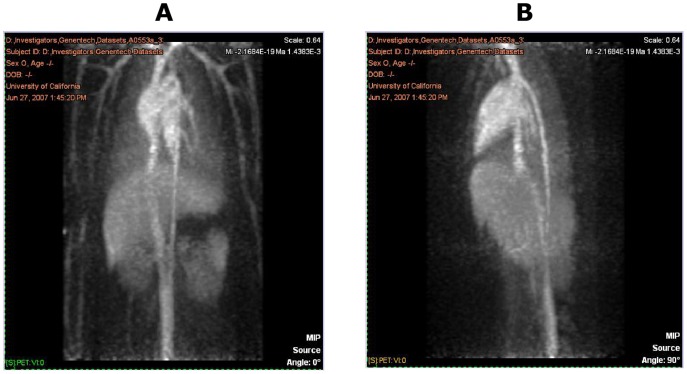
PET Images from One Representative Animal at 1 hour Post Dose: Frontal View (A) and Sagittal View (B).

**Figure 5:**
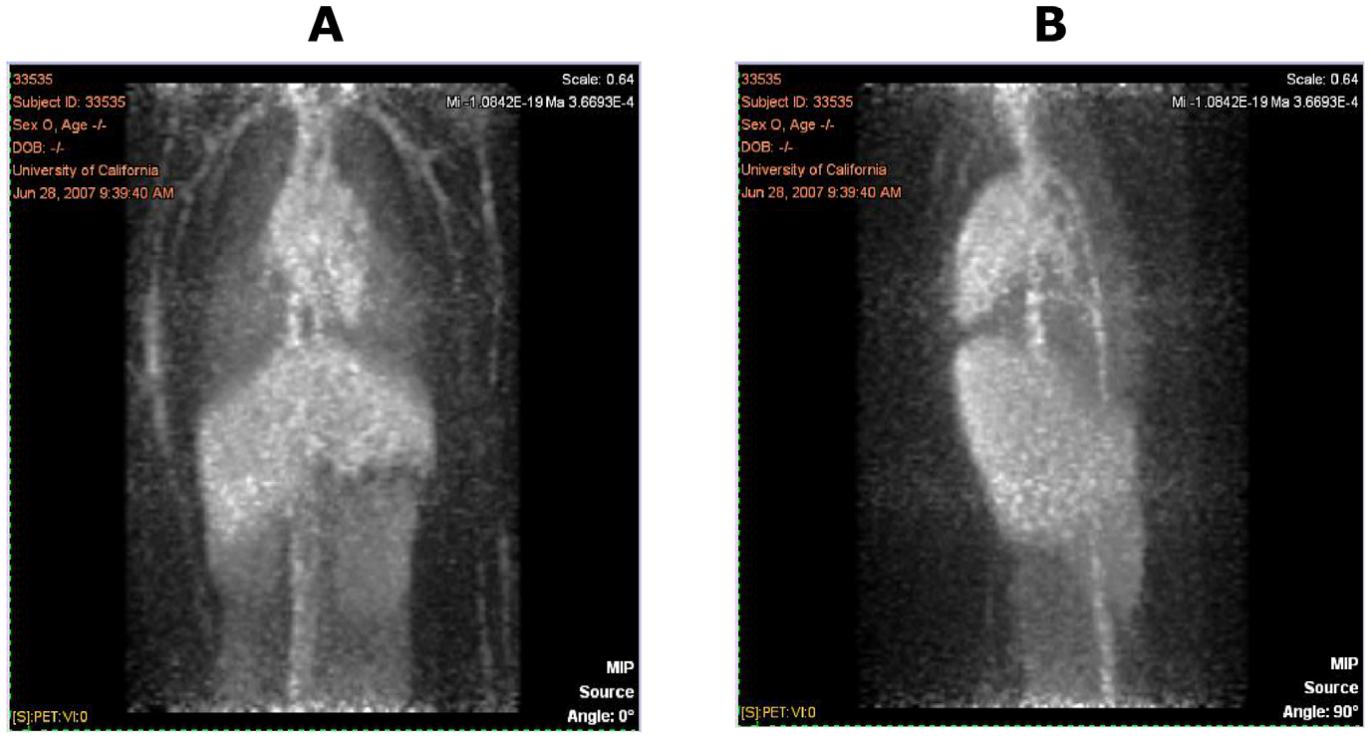
PET Images from One Representative Animal at 24 hours Post Dose: Frontal View (A) and Sagittal View (B).

**Figure 6:**
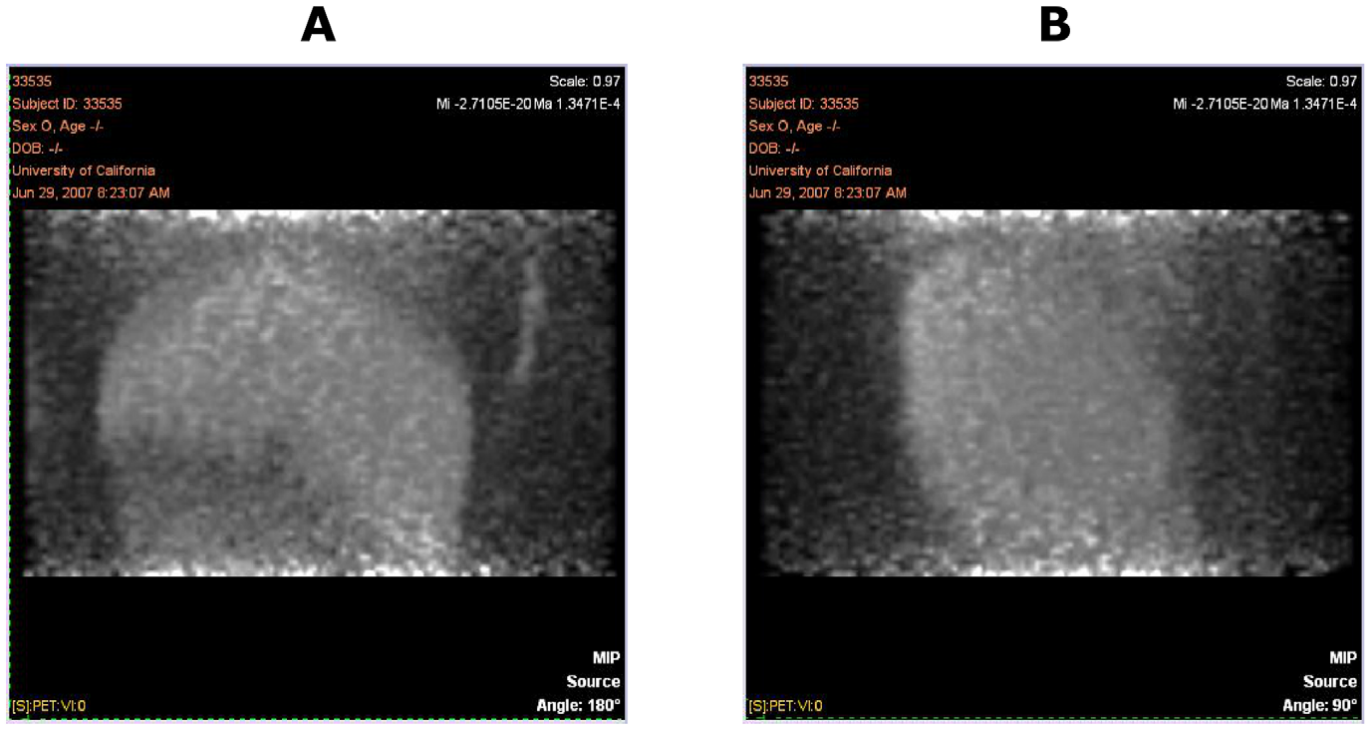
PET Images Showing a Limited Field of View (Mainly Liver) from One Representative Animal at 48 hours Post Dose: Frontal View (A) and Sagittal View (B).

PET images from one representative animal at the various time points are shown in Figures 4, 5, and 6. These figures show the frontal (coronal) and sagittal maximum intensity projections of the full extent of the images. The images cover areas from approximately the neck to the pelvis, with the exception of the 48-hour scans, which were limited to the central trunk (mainly liver). The image intensities are approximately proportional to the amount of radioactivity (and therefore anti-oxLDL) present in a given 3D voxel. All images demonstrate the expected minor artifacts resulting from the non-overlapping combination of the three separate bed positions. These artifacts are evident as horizontal bands of somewhat increased noise dividing the images into three equal parts, and in some instances can cause the vena cava to appear to be discontinuous.

The images obtained at the 1-hour post-dose timepoint showed distribution predominantly to the blood pool, typical for an antibody that has no large and rapid antigen sink. Chambers of the heart and major vessels of the thorax, abdomen, kidneys, lungs, and limbs are visible, along with diffuse blood pool signals in liver, spleen, and bone marrow. In the images obtained at 24 hours, this pattern was maintained, typical of an antibody with a long half-life and no significant site of rapid uptake. There were no tissues showing rapid uptake, and at 24 hours post-injection the antibody remained predominantly in the blood pool. No uptake into the spinal cord was observed, nor any uptake into the intestines or other abdominal organs. At 24 hours post-dose, 2 animals displayed a small area of focal uptake dorsal to the heart, in what appeared to be the descending thoracic aorta (data not shown). The significance of this is unknown, and likely to be an artifact, although it is consistent with accumulation of signal at the site of atherosclerotic plaque.

## Discussion

Anti-oxLDL is a novel antibody targeting MDA-oxidized human ApoB-100. Anti-oxLDL has been shown to reduce plaque formation in in vivo mouse models of atherosclerosis [3], [7]. We are interested in developing anti-oxLDL due to its therapeutic potential in the secondary prevention of major cardiac events in high risk patients with ACS. To gain a better understanding of the PK and disposition of anti-oxLDL, this study investigated its PK and tissue biodistribution in cynomolgus monkeys using PET imaging.

ELISA results indicated that binding of DOTA-anti-oxLDL to ApoB100 may have been slightly reduced, compared with unconjugated anti-oxLDL, although it is unlikely that such a minor reduction would have affected imaging quality. From our previous positive experience using DOTA conjugates for imaging, we could reasonably conclude that the antigen binding affinity of the DOTA-anti-oxLDL conjugate used for imaging was not materially different from that of unconjugated anti-oxLDL.

Serum concentrations of anti-oxLDL were measured using two ELISA methods measuring free (unbound and partially bound) anti-oxLDL and total anti-oxLDL. The free concentration would include both the unbound and partially bound (i.e., monovalently bound) forms due to the bivalency of the monoclonal antibody, while the total concentration would include the free (unbound and partially bound) as well as the bound forms [10]. Serum concentrations and PK parameters from the two ELISA methods were fairly similar suggesting that the systemic clearance of total anti-oxLDL might be similar to that of free (unbound and partially bound) anti-oxLDL. Anti-oxLDL clearance values were slightly higher than typical IgG1 antibodies in cynomolgus monkeys [11]. In addition, there were no gender differences in pharmacokinetics of anti-oxLDL in cynomolgus monkeys. From the biphasic PK profile of anti-oxLDL, there did not appear to be any evidence of target mediated drug disposition. This was expected as the concentrations of oxLDL in normal healthy cynomolgus monkeys (like the ones in this study), are much lower than in hypercholesterolemic cynomolgus moneys [12]. However, the concentrations of oxLDL were not measured in the monkeys in this study and further studies looking at the pharmacokinetics of anti-oxLDL in animal models with high levels of oxLDL may be needed to assess the impact of oxLDL concentrations on the PK of anti-oxLDL.

While PET imaging to investigate tissue distribution of monocolonal antibodies has been used extensively in rodents [13], to our knowledge this study is one of the first to use this technique in cynomolgus monkeys. PET imaging showed that the distribution of anti-oxLDL in cynomolgus monkeys is predominantly to the blood pool, visible as the heart and great vessels in the trunk and limbs, plus diffuse signals in the liver, kidney, spleen, and bone marrow. This biodistribution pattern appears to be typical of an antibody that has no large, rapid antigen sink outside the blood space. This finding is consistent with the fact that the target antigen for anti-oxLDL is in the blood compartment. However, from the PK results, the volume of distribution at steady state was 3-fold higher than the serum volume suggesting some extravascular distribution. One explanation for this discrepancy could be the limitation of the PET imaging technique given the short half-life of the ^64^Cu radioisotope (∼12 h) compared to the relatively longer distribution half-life of anti-oxLDL (∼20 h). Since the imaging time points were within a 48 hour window, it may not have been long enough to see accumulation of anti-oxLDL in the tissues. Another possible explanation could be that anti-oxLDL distributes to the cells lining the blood vessels and the PET imaging technique is not sensitive enough to distinguish between the blood compartment and the lining of the blood vessels. Further studies using more sensitive techniques may be needed to evaluate the distribution of anti-oxLDL to the lining of the blood vessels and at longer time points in the tissues. In addition, studies in a disease model of atherosclerosis may be useful to understand the distribution of anti-oxLDL within the atherosclerotic lesions.

In summary, the PK and tissue distribution of anti-oxLDL were characterized in cynomolgus monkeys. The clearance of anti-oxLDL was slightly higher than typical IgG1 antibodies in cynomolgus monkeys and its biodistribution pattern appears to be consistent with an antibody that has no large, rapid antigen sink outside the blood space.
